# Pufendorf and Leibniz on duties of esteem in diplomatic relations

**DOI:** 10.1177/17550882211002225

**Published:** 2021-03-18

**Authors:** Andreas Blank

**Affiliations:** Alpen-Adria-Universität Klagenfurt, Austria

**Keywords:** Ambassadorial rights, dependence, esteem, natural law

## Abstract

The striving for self-worth is recognized as a driving force in international relations; but if self-worth is understood as a function of status in a power hierarchy, this striving often is a source of anxiety and conflict over status. The quasi-international relations within the early modern German Empire have prompted seventeenth-century natural law theorists such as Samuel Pufendorf and Gottfried Wilhelm Leibniz to reflect about this problem. In his *De statu imperii Germanici* (1667), Pufendorf regards the power differences and dependencies between the Reichsstände to be an expression of the deficits of constitutional structure of the Empire—a structure that, in his view, causes internal division because it leads to distorted practices of esteem between the estates. Against Pufendorf, Leibniz argues De jure suprematus ac legationis (1671) that political actors such as the German princes who are not Electors could fulfill functions under the law of nations such as forming confederations and peace keeping. Incoherently, however, Leibniz excludes less powerful estates such as the Imperial cities and the Hanseatic cities from the ensuing duties of esteem. This shortcoming, in turn, is arguably remedied in Pufendorf’s later considerations concerning duties of esteem in diplomatic relations.

## Introduction

To some, it may seem inevitable to think of esteem in international relations as a function of power—those nations with more power will make claims to higher esteem. Since such claims are the outcome of highly competitive politics, they will be the origin of conflict and status-insecurity of the less powerful nations. One dimension in which esteem for political communities expresses itself is the realm of diplomacy, where questions of protocol, precedence, and rank always has played a significant role. The question of esteem in diplomatic relations is particularly pressing with respect to political actors and communities that lack independence but nevertheless fulfill functions of civil government. While such muddled constitutional situations have not disappeared from the planet, they were pervasive in the early modern German Empire. Of course, this situation has been the object of studies in constitutional history for many decades. However, as far as I can see, the consequences of this constitutional situation for the dynamics of esteem between political communities in early modern Germany have not been explored in detail. Samuel Pufendorf (1632–1694) and Gottfried Wilhelm Leibniz (1646–1716) have given thoughtful considerations to the problems arising from the power-oriented competition for the dynamic of esteem within the German Empire, and their considerations deserve attention since both Pufendorf and Leibniz give illuminating clues as to how respecting the desire for esteem in diplomatic relations can play a *stabilizing* role both within the Empire and in European politics.

Since both Pufendorf and Leibniz refer to particularities of early modern German public law, it may be useful to indicate very briefly how the institutional structure of the German Empire exemplified two central aspects of a constitutional order: the rule of law and the separation of powers.^1^ While there was no single legal document that defined the institutional framework, law played a role through a multiplicity of so-called fundamental laws (such as the Bulla Aurea of 1361), a long series of so-called capitulations that defined the conditions under which an Emperor was elected, and a complex web of privileges given by the Emperor to the political entities constituting the Empire (the *Reichsstände*)—privileges that were revocable at the beginning but gradually turned into incontestable rights. Often these legal documents did not create rights and duties that did not exist before; rather, they made aspects of previously accepted customary law explicit. Customary law, in turn, was often regarded to be an expression of natural law. This is why the legal codifications of the institutional structure of the Empire were believed to uphold public security, to enable peaceful conflict resolution and to defend the natural rights both of individuals and of political communities.

To promote these objectives, the legal framework introduced a sophisticated system of distribution and separation of powers, but at the same it led to a situation in which no single political body and no single political actor was fully independent. For instance, the Emperor depended on the Electors not only in the moment of the election but also during the entire duration of his reign due to the obligations laid down in the capitulations. Each single estate depended on the legislation and other decisions of the Imperial Diet (the *Reichstag*)—the representation of all estates—in matters that concerned the Empire. The Diet had the right of proclaiming the *Reichsacht* (a kind of impeachment) against representatives of estates who violated their duties of loyalty to the Empire. And the estates were obliged to cooperate with the imperial institutions for upholding public peace—a purpose for which the compliance mechanism of the *Reichskreise* was established. Each *Reichskreis* consisted of a group of estates that, acting as a single political body under unified leadership, was powerful enough to fulfill an executive function (*Reichsexekution*) with respect to each of its members.

Unlike the Electoral courts, the jurisdiction of the princes and the Imperial Cities was not exempt from appeals to the imperial courts—an arrangement that allowed not only to overturn the decisions of the territorial courts but also to deal with lawsuits of citizens against their territorial authorities and to decide controversies between estates. There was not only one such court but two of them, the *Reichkammergericht* and the *Reichshofrat*, the former working with judges nominated by the estates, the latter working with judges nominated the Emperor and supervised by delegates of the Elector of Mainz. At the same time, the larger territories had wide-ranging independence in administrative matters, in raising taxes, in forming armies, and in adopting confessional politics. Only the Imperial Cities were happy to accept interference of the Emperor into their administrative structure and their republican decision procedures since accepting this kind of dependence was understood to be an effective defense of their independence against the princes’ striving for territorial consolidation.

In late seventeenth-century Germany, perhaps no-one took a more critical stance on constitutional realities than Samuel Pufendorf.^2^ In *De statu imperii Germanici* (1667), Pufendorf argues that one of the detrimental consequences of the competition between dependent political communities with large differences in power and political structure consists in a negative dynamic of esteem—a dynamic in which contempt contributes to exacerbating the antagonisms among the *Reichsstände* and between the *Reichsstände* and the Emperor. In *De jure suprematus ac legationis* (1671)—partly written in response to Pufendorf’s *De statu imperii Germanici*—Leibniz suggests that a normative conception of duties of esteem in diplomatic relations could offer a remedy to the antagonisms inherent in the constitutional structure of the Empire. Puzzlingly, however, he offers this solution only with respect to those German princes who are not Electors, thereby excluding smaller political communities such as the partly overlapping groups of Imperial Cities and Hanseatic cities. In his *De jure naturae et gentium* (1672, expanded second edition 1684), Pufendorf gives a general, natural law-based account of how duties of esteem in diplomatic relations could be reconceptualized in such a way as to become proportional to the fulfillment of the goals of civil government rather than proportional to power. And in his *Sechs und Zwantzig Bücher der Schwedisch- und Deutschen Kriegsgeschichte* (1688), he documents that the Hanseatic League not only fulfilled functions under the law of nations but also made normative claims to ambassadorial rights based on the fulfillment of these functions.

To set the stage, it will be useful to draw attention to a series of remarks concerning problems caused by disesteem in Pufendorf’s analysis of the constitution of the Empire (section 2). Pufendorf’s diagnosis will help to make clear the relevance of Leibniz’s arguments that purport to show that that the role of the German princes is not as dysfunctional as Pufendorf has thought, and that this role both grounds duties of esteem in diplomatic relations and is facilitated by the fulfillment of these duties of esteem (section 3). However, this is only a partial response to Pufendorf’s concerns since Pufendorf had not only the German princes in mind but also other *Reichsstände* such as the German cities; by contrast, Leibniz excludes the German cities from the realm of bearers of diplomatic rights (section 4). Arguably, Pufendorf’s normative accounts of duties of esteem in diplomatic relations offers a more coherent answer to the problems of a power-oriented conception of status in diplomatic relations (section 5). Pufendorf’s historical observations concerning the role of the Hanseatic League in European diplomacy suggests that the scope of these duties could include even small, dependent communities such as the Hanseatic cities (section 6).

## Pufendorf on esteem and disesteem in the constitution of the German Empire

The central topic of Pufendorf’s *De statu imperii Germanici* is the genesis of a constitutional situation where sovereignty rights are distributed across hundreds of *Reichsstände*.^3^ As Pufendorf argues, this situation has many detrimental consequences, such as the aggravation of confessional differences, the lack of a unified system of taxation, the lack of a coordinated system of military defense, the lack of coherent legal codification and the persistence of a system of overlapping and competing spheres of jurisdiction. These are all topics that have been carefully discussed in Pufendorf scholarship. However, what has not found much attention, as far as I can determine, are Pufendorf’s remarks about how this constitutional situation affects the dynamic of esteem.

Pufendorf’s attitude toward the dynamic of esteem in political life is ambivalent. As far as the medieval constitution of the German Empire goes, Pufendorf believes that the desire for esteem had played a stabilizing function in the late medieval period. This becomes clear in his assessment of the role of capitulations: “[I]n the capitulations, the Electors prescribe to the Emperor what he should do and what he should avoid; *but not as if they had some power over him but rather in the way of contract* . . .” (De Monzambano [Pufendorf] 1667: 184). Of course, such a contract without the power of sanctioning its violation raises the question of the origin of the binding force of the agreement. As Pufendorf notes, according to the *Bulla Aurea* the *comes palatinus* had the function of deciding controversies concerning whether the Emperor fulfilled the conditions of capitulation.

Pufendorf conjectures that this institution had its origin in the times when the *comes palatinus* at the same time fulfilled the function of a *majordomus* at the royal court—that is, a function that in no way represented a power independent of the court. The binding power of the decisions of the *comes palatinus* thus could not derive from anything like a sanctioning power but rather from the attitude that the king took toward a legal authority: “The king adhered to the sentence of the *comes palatinus*, not as if he recognized a superior power, but because once he knows the right of the one who submitted the demand, he cannot fail to fulfill his obligation” (1667: 185). Pufendorf compares the dynamics behind the origin of this obligation to the practice of German princes to let questions concerning their own duties be decided by their own courts: “these courts, however, cannot at all force a prince, or exert much coercion, if the reverence for the right, for conscience and for public esteem would not motivate him to fulfill his duties” (1667: 186). This comparison indicates that, what in Pufendorf’s view, made the contracts constitutive of limited monarchy work were moral qualities of the ruler—his respect of law and conscience—as well as his desire for public esteem.

However, Pufendorf notes that, in the modern period, this function of the *comes palatinus* has been abandoned (1667: 185). Hence, also the dynamics on which limited monarchy was previously based cannot find an institutionalized setting. On the contrary, Pufendorf believes that these elements of limited monarchy are undermined by two opposing tendencies of the *Reichsstände*: (1) their claim to independent jurisdiction; and (2) their claim to the right confederation without consulting the Emperor (1667: 188). He argues that this situation distinguishes the constitution of the German Empire from limited monarchies (1667: 188). In his view, feudal relations have thereby turned into relations that hardly differ from confederations between unequal partners (1667: 77). He takes it to be a genuine possibility that the German territories could develop into a comprehensive system of confederation, in which the *Reichsstände* would mutually support each other and at the same time maintain reverence (*reverentia*) toward the Emperor (1667: 190).^4^ However, in order for such a system to be stable, Pufendorf argues, confederation has to be useful for all confederates; and this can be only the case if the confederates are not characterized by too different constitutions and too large power differences (1667: 211).

One aspect that, in his view, is detrimental about this in-between situation is that it leads to a *negative* dynamic of esteem. As long as such differences in power and inner constitution persist, Pufendorf assumes, there will be an antagonism (*renitentia*) among the *Reichsstände* and between the *Reichsstände* and the Emperor. If so, then the estates’ tendency toward independent jurisdiction and independent confederation have the consequence that the reverence (*reverentia*) expressed toward the Emperor consist in “inert images” (*inania simulacra*) (1667: 188)—which could explain why the *Reichsstände* are less inclined to let their politics be guided toward political goals defined by the Emperor. These tendencies are also connected with the development of contempt between the single *Reichsstände*:

It has been observed for a long time that monarchies hardly form confederations with republics in good faith, not even temporarily, nor are they suitable to form perpetual confederations. For princes have an aversion against plebeian liberty, and the people abhor the haughtiness of princes. Probably this is the perversity of the human genius that it hardly can bear with a quiet mind to see someone enjoying the same right although he possesses visibly inferior power. (1667: 211–212)

From this dynamic of contempt for those communities that have different constitutions or less power, Pufendorf maintains, a situation arises that is characterized by perpetual suspicions, distrust, and hidden intrigues that have the aim of preventing other *Reichsstände* from becoming more powerful or of diminishing the power that they already have (1667: 212)—a situation in which envy and contempt often express themselves in open insults (1667: 214) and in the desire to oppress the less powerful (1667: 215). In this way, the dynamics of disesteem exacerbates the disintegrating tendencies of the striving for independent jurisdiction and independent confederation characteristic for the politics of the estates.

## Leibniz on esteem and diplomatic rights of the German princes

While for Pufendorf the question of esteem and disesteem between estates seems to have been a side-issue in his analysis of the constitution of the German Empire, this question becomes central in Leibniz’s *De jure suprematus ac legationis*. Like many works of political theory in the early modern period, Leibniz’s text is partly motivated by a concrete political agenda—in this case the support of the right of Duke Johann Friedrich von Braunschweig-Lüneburg to have his envoys be recognized as ambassadors. At the same time, Leibniz approaches this task in the framework of a serious theoretical discussion of the nature of sovereignty rights, meant in part to be a response to Pufendorf’s emphasis on dysfunctional aspects of the constitution of the German Empire.^5^ Arguably, this dual context of Leibniz’s work explains some unresolved tensions between his treatment of diplomatic rights of the German princes and his treatment of the diplomatic rights of the German cities.

The importance that political thinkers in early modern Germany conferred to the question of whether the German princes who are not Electors have the right to demand that their envoys be recognized as ambassadors may easily elude present-day readers. After all, the practical consequences of the distinction between ambassadors and lower-level envoys were restricted to matters of protocol, that is, to ceremonial matters and titles. This limited range of practical consequences, as Leibniz makes clear, is due to the fact that other relevant distinctions of diplomatic function—for example, the distinction between holding a permanent office and being sent to take care of a particular negotiation, or the distinction between having the full power to sign agreements and being obliged to consult the prince before signing agreements—do not coincide with the distinction between being an ambassador and being a lower-level envoy (1923: IV, 2: 40). Still, the difference between the different types of emissaries was seen to be significant. As Leibniz (1923) emphasizes, only ambassadors were regarded as having “representative character” (*character repraesentatitium*) (IV, 2: 42). That is to say that they not only fulfill a certain function in the process of negotiation but are also regarded as placeholders who have the right to be treated, within the limits of custom, as if the persons whose place they hold were present (1923: IV, 2: 43). This is why matters of protocol became so important: they were regarded as external indications of whether the emissary was regarded as the representative of a sovereign or not.

Leibniz (1923) explains that the question of whether the German princes have the right to send ambassadors is a question of honor and at the same time a question of justice. More specifically, he treats the question of the diplomatic rank of the envoys of the German princes as a matter of “distributive justice with respect to honor and rewards” (*justitia distributiva quae circa honores et praemia versatur*) (IV, 2: 141). In particular, he treats the right to send ambassadors as a matter of treating like cases in like manner—a central component of the idea of justice:

The same signs of honor have to be shown toward him and to his envoys that are shown to all those who use the same right of weapons, *for the law of peoples does not permit that those are treated differently, due to lack of respect, who have in hand the same cause as the others by means of which they vindicate for themselves the privileges of the law of peoples*. (1923: IV, 2: 140; Leibniz’s emphasis)

Thus, what equates the German princes to the great European powers is their recognized right to engage in warfare that they share with the great powers. Consequently, “whatever is conceded to many on the basis of some ordinary rights, such that it is also demanded by them, cannot be denied without injustice to those others who possess the same dignity” (1923: IV, 2: 141).

To defend this line of thought, Leibniz (1923) considers an objection according to which matters of honor and rewards, very much like alms to the poor, should be regarded as matters of generosity rather than justice, so that neglecting duties of providing honor and rewards does not give rise to legal action (IV, 2: 141–142). He responds: “[T]his is not so when honor is paid to princes . . . For someone who shows reverence to someone in a position of great dignity, does not give something that is owed out of generosity but does what is his duty” (1923: IV, 2: 142). As he argues, this is so because the one who refuses to show the external signs of honor expresses contempt: “From whence arises resentment, the desire for vengeance, and, at last, war which the task of the law of peoples is to avoid” (1923: IV, 2: 142). Here a second specifically political aspect of honor comes into play: refusing to respect the signs of honor is perceived as an insult, with all the conflict potential that the passions triggered in this way bring with them. This is why matters of honor influence decisions concerning war and peace. Consequently, “for the more cultivated nations of Europe, the care for dignity forms part of the law of peoples” (1923: IV, 2: 142).

Three interrelated requirements, Leibniz (1923) concludes, are therefore essential in peace negotiations: (1) that the dignity of the parties in conflict be respected; (2) that some way be found in which the passions of the parties in conflict can be made innocuous for the course of negotiations; and (3) that as far as honor is concerned, interests do not matter, but rather justice (IV, 2: 144). Recognizing the dignity of envoys certainly contributes to moderating passions, and will do so only if it is done not out of arbitrary interest but with the intention of acknowledging the right of envoys to be recognized as ambassadors and the right of the powers to be recognized as sovereigns (1923: IV, 2: 144).

Still, this line of argument rests on the assumption that the German princes have the same diplomatic rights as the great European powers and, on first sight, this assumption seems to disregard the enormous power differences between the great European monarchies and the smaller principalities. To dispel this impression, the main argumentative thrust of *De jure suprematus ac legationis* goes in the direction of establishing the relevant sense of likeness between the great monarchies and the territories of the German princes, which bestows the same diplomatic rights to all of them. As to historical fact, Leibniz (1923) refers to Bodin’s list of alliances of German princes with powers outside the Empire (IV, 2: 101; see Bodin, 1576: 1.7). As to the question of right, Leibniz argues that the German princes fulfill functions according to the law of peoples:

The rights of princes that relate to others and beyond the territory are the right of war and peace, the right of confederation, the right of entertaining an army, . . . the right of sanctions against those who are not members of the Empire, . . . the right of intervening in general treaties and councils, . . . the right to offer mediation, to accept arbitration, to promise guarantees, the right to assist the oppressed even if they are foreigners. (1923: IV, 2: 94)

Leibniz does not regard the constitutional structure of the German Empire to be ideal. But he regards alliances within the Empire as an emergency solution resulting from the political weakness of the Empire’s institutions:

If our community were well constituted, . . . if the weak were safe under the law from oppression by the powerful, I concede that there would be no need for dangerous remedies such as extraordinary alliances. But who would not see that in the current confusion of the political situation, it is thanks to the alliances that we still have a community? In this way, alliances between some *Reichsstände* once suppressed the threats to public peace, such as robber castles, and laid the foundation for the imperial constitution that has continued to exist since Emperor Maximilian I. (1923: IV, 2: 101–102)

Leibniz thus believes that the constitutional structure of the Empire came into being *because* the institutions of the Empire were too weak to guarantee justice and security. From this perspective, Leibniz uses the capacity of fulfilling the function of mediation involving powers outside the Empire as an argument in favor of the sovereignty of the German princes. Such mediation provides an instance in which the German princes can effectively intervene in international conflict resolution, and because this is an instance of an activity that combines the capacity of using military force with the obligation to respect demands of justice, these activities count as examples of the political agency of sovereigns, even if the actors are not independent from the institutions of the Empire in other respects.

This is why Leibniz applies to the envoys of the German princes the insight that the esteem in which ambassadors are held to be an expression of natural reason (and thus of natural law):

[I]t is customary to say that ambassadors are inviolable. For he who is sent to conduct negotiations enjoys immunity accepted by the law of peoples and introduced by natural reason . . . For natural reason demands that the capacity of local movement be free; otherwise, if this possibility of building trust ceases, there is no cure for the sliding into the festering of rational souls. (1923: IV, 2: 38)

Leibniz (1923) emphasizes that the inviolability of ambassadors includes not only protection against physical damage, but also protection against defamation (*contumelia*) (IV, 2: 38). As he clarifies, honor consists in opinion and habits, where the relevant opinion can be described as an assessment of dignity (*aestimatio dignitatis*) (1923: IV, 2: 142). He also explains why violations of honor against sovereigns and their ambassadors belong to the law of peoples: such violations of honor lead to a hostile attitude and to the desire for retaliation, and thus ultimately to wars; and it is the goal of the law of peoples to prevent wars (1923: IV, 2: 142). He observes that the refusal to recognize envoys as ambassadors can also delay a peace agreement, as can be observed in the protracted negotiations that preceded the Peace of Westphalia. There, the refusal to recognize the envoys of the house of Brunswick as ambassadors brought the peace negotiations to a halt (1923: IV, 2: 353–354). Conversely, Leibniz (1923) argues that respect for equal rights among sovereign communities is one of the foundations of universal peace because it avoids sowing the seeds of new hatred (IV, 2: 142).

What Leibniz (1923) has in mind is not only that withholding certain forms of esteem from the envoys of German princes has unfavorably practical consequences; rather, his point is that these consequences concern the fundamental issue of the law of peoples. This becomes clear when he uses the natural-law-based function of preserving security and peace as one of the criteria for what belongs to the realm of the law of peoples (IV, 2: 18). In this sense, the fulfillment of duties of esteem toward the representatives of the German princes is essential for the fulfillment of the function of their representatives under the law of peoples. This is why the duties of esteem toward the envoys of the German princes have a foundation in natural law because they play a role in upholding security and peace.

## The question of diplomatic rights of German cities

With a view to the German princes, Leibniz’s argument thus provides the resources required for answering Pufendorf’s diagnosis that the constitutional situation of the Empire leads to contempt for less powerful *Reichsstände*. Leibniz provides reasons for why the same rights, such as confederations rights, should be conceded to political agents with different power positions—in fact, even to agents in a dependent position. Also, he provides reason for why these rights also give rise to duties of esteem in diplomatic relations. At the same time, Leibniz excludes Imperial Cities and other Hanseatic cities from the realm of bearers of diplomatic rights. Is this position coherent?

Heinrich Henniges (1645–1711), one of Leibniz’s critics, challenged the coherence of this position. Henniges was a professor of law in Frankfurt/Oder and worked as a consultant and diplomat in the service of the Elector of Brandenburg. Therefore, it is not surprising to see that he defends the exclusive embassy rights of the Electors. In the course of this defense, he develops the objection that Leibniz does not adequately explain where the boundary between sovereignty (*suprematus*) and territorial dominion (*superioritas*) lies. As Leibniz (1923) understands the distinction, territorial dominion is limited to jurisdiction and the ability to suppress a revolt of subjects by military force (IV, 2: 18; 56), while sovereignty includes the ability to influence other political communities in several ways: through military means, through non-military sanctions, and through participation in international negotiations (1923: IV, 2: 18; 56–57).

Henniges takes the problem of defining the border between sovereignty and territorial dominion to be serious. For if one considers the variables mentioned by Leibniz—size of territory, tax revenue, financial means to maintain an army, the practice of forming alliances, and the practice of sending envoys—then cities (such as Hamburg, Nuremberg, Frankfurt, Lübeck, Ulm, and Cologne) and Imperial Knights (like the Counts of Oldenburg) are in a comparable position. In this case, it would be incoherent from Leibniz’s point of view to deny sovereignty to these *Reichsstände* (Henniges, 1687: 63–66). Thus, if, the princes who are not Electors are conceded the right to send ambassadors, then the same right cannot be denied to the cities and Imperial Knights. Of course, both Leibniz and Henniges reject this consequence. Therefore, Henniges’s argument can be understood as a *reductio ad absurdum*. But is the consequence really as absurd as it seemed to Henniges?

Leibniz (1923) argues that the purpose of the medieval city alliances was to secure routes and trade relations (IV, 2: 100). However, this argument does not capture adequately the intricacies of seventeenth-century views concerning the legal status of the German cities. This can be seen in a dissertation directed by Hermann Conring (1606–1681), who, like Leibniz in the period of *De jure suprematus ac legationis*, was active in several functions for the house of Brunswick, and with whom Leibniz corresponded during this time (see 1923: II, 1: N. 150; N. 151). Conring and Bode (1652) point out that the Hanseatic cities received immunities and other trade privileges from their trade partners in the Nordic countries (1652: § 99). They argue that the Hanseatic League was a means to defend immunities and other rights against violence and injustice (1652: § 100). Conring and Bode (1652) note that a further legal argument for the legitimacy of the Hanseatic League derives from the consideration that the League served as a protection of trade rights against piracy (§ 100).

Still, Leibniz (1923) does not assign normative power to the fact that, in 1570, an alliance was formed between the Hanseatic city of Lübeck and the Danish King Frederick II (IV, 2: 101). Leibniz (1923) argues that the Hanseatic cities have no sovereignty because some of them are not free Imperial Cities, and thus are dependent on intermediary powers (IV, 2: 62). Here, Leibniz seems to commit a *petitio principii*, for it is precisely the question whether the difference in constitutional rank implies that Hanseatic cities cannot acquire sovereignty rights on the basis of natural law. An additional argument is needed, and in fact Leibniz (1923) argues that communities can possess sovereignty rights only if they can influence the highest European politics through military power and alliances, which, in Leibniz’s view, is not the case with the Hanseatic cities (IV, 2: 18). Leibniz (1923) argues that even Imperial Cities do not possess sovereignty rights because they can only wage war in alliances with other *Reichsstände* (IV, 2: 18).

Against this line of argument, it could be objected that it is precisely through alliances that smaller powers seem to have opportunities to exert a degree of influence on European politics, and that not all such influences need to be bellicose. The role of the Hanseatic League in upholding peace is important because it is central for an argument in favor of the legitimacy of city alliances from the perspective of the law of peoples. For instance, Conring and Bode (1652) use this argument to counter a possible objection against the legitimacy of the Hanseatic League. According to this objection, the confederation of the Hanseatic cities caused damage to the Empire because the confederation was without any public authority of the Empire; rather, it was formed on the private initiative of dependent cities (§ 100). It also caused damage to the Hanseatic cities themselves, the objection goes on, because being destitute of the authority of the Empire, they had to repel the violence of external powers all of their own (1652: § 100). And Conring and Bode (1652) concede that such a development “is not at all to be tolerated in a well-formed commonwealth” (§ 100).

The question, however, is whether the German Empire had at any time been well-formed enough to provide the necessary protection of trade rights and inner security. For Conring and Bode (1652), it is exactly the weakness of the constitutional structure of the Empire that makes city alliances legitimate. As they argue, confederations such as the Hanseatic League were useful for the entire Empire not just in the sense that they were responsible for the affluence of commercial goods: “[F]or several centuries, we owe to them public cohesion and the tranquility and the liberty of the people. Even if they may sometimes have transgressed their limits, if we want to say the truth, without this remedy all would have been oppressed by extreme servitude” (§ 104).

In this respect, Leibniz’s (1923) own views are so close to Conring and Bode’s that it is puzzling to see that he resisted the conclusion that city alliances can be legitimate bearers of diplomatic rights. Leibniz is aware that the *Bulla Aurea* of Emperor Charles IV allows the princes and cities to form alliances whose goal is the general peace of the provinces and territories (IV, 2: 101). Leibniz (1923) also points out that this constitutional basis of confederation rights had in the meantime found decisive confirmation under international law, for the Peace of Westphalia (1648) ascribes to all *Reichsstände* the right to form alliances, both among themselves and with external powers, for their own preservation and security, provided that these alliances do not harm the peace in the Empire (IV, 2: 102).^6^ Leibniz (1923) cannot therefore have overlooked the fact that the right of alliances between cities is supported by the fundamental laws of the Empire. With regard to the Reformation period, Leibniz points out that religious peace was only achieved through alliances. Likewise, he argues that the freedom of the Empire against French and Spanish claims to power could only be defended through alliances (IV, 2: 102). Moreover, he compares the peace-keeping functions of the medieval city alliances with the peace-keeping functions of confederations between the German princes (1923: IV, 2: 102). This makes it difficult to understand why, if the peace-keeping functions of confederations of princes are counted as belonging to the law of peoples, as Leibniz does, the peace-keeping functions of city alliances should be excluded from this realm. Of course, excluding the Hanseatic cities from ambassadorial rights is fully coherent with Leibniz’s support of the political agenda of the Guelfs; but it is not fully coherent with the theoretical principles that Leibniz uses to build his argument.

## Pufendorf on duties of esteem between political communities

Does Pufendorf offer a more coherent theoretical account of the nature and foundations of duties of esteem in diplomatic relations? Arguably, he does. His normative treatment of duties of esteem between political communities does not go into the details of constitutional law and political history. But it certainly derives its interest from the problems that power-oriented conceptions of esteem create in political reality. Pufendorf’s treatment of duties of esteem in *De jure naturae et gentium* is complex and comprises at least three different themes: (1) duties of esteem based on commonly shared natural rights and natural duties (Pufendorf, 1684: 5.2.1–7; see Hruschka, 2000: 191–193; Saastamoinen, 2010); (2) duties of esteem based on the degree of the fulfillment of duties of natural law and of the obeyance of positive laws (1684: 8.4.2–10; see Haara and Lahdenranta, 2018; Haara, 2018: 120–125; Haara & Stuart-Buttle, 2019: 709–714); and (3) duties of esteem based on the political agency of sovereigns in defining a hierarchically structured order of social positions (1684: 8.4.11–32). Here, I will be concerned with the third theme—a theme that has not, as I far as I can determine, found much sustained attention from commentators. This is unfortunate because Pufendorf’s remarks concerning esteem toward the representatives of other political communities occur in the context of the discussion of the role of sovereigns in determining the value of subjects.

Pufendorf’s architectonic decision is telling because it implies that, for him, esteem between political communities and their representatives is as much a matter political decision making as is the definition of the particular order of social ranks. It is not difficult to see why Pufendorf maintains that political agency determines the duties of esteem between political communities. After all, the matters of diplomatic protocol that express relations of precedence between political communities and their representatives are a matter of political decision making. Precedence between political communities seems to be nothing other than ascribing to communities a certain order of rank. Still, Pufendorf contests the view that such acts of assigning rank should follow a hierarchy of power. He does so although he shares Hobbes’s view that esteem functions in a way analogous to prices and that, therefore, it is always others that determine one’s worth:

*Intensive Esteem* is that Species of Reputation, by which Persons, otherwise equal in the *simple Repute*, are preferr’d to one another, according as one possesses a larger Share than *another of those Things*, whatever they be, which are apt to raise in other Men *Reverence* and *Respect*. And therefore the *Honour* is not really in him that *receives*, but in him that *gives it.* For though a Man may set what *Value* he pleases upon himself, as the *Seller* does upon his *Commodities*: Yet, as in these, it is the *Buyer* at last that determines the *Price*; so the *Value* of Men is no higher or greater, than as others are pleas’d to set it. For as *Hobbes* says, *Let a Man (as most Men do) rate himself at the highest he can; yet the true Value of him is no more than he is esteem’d at by others*. (1729: 805 [8.4.11]; see Hobbes, 2012: 10.16)

A further view that Pufendorf takes over from Hobbes is the insight that, if the worth of a person is determined by others, then esteem is essentially comparative. Pufendorf (1729) cites approvingly the following passage from *De cive* (p. 805):

It is impossible any *Society* should be either great, or of any long Continuance, that at first united only upon such a *Bottom* as a *common* Desire of *Glory*, or upon mutual Obligation to assist one another in their Pursuits after *honor*; because *Glory*, and the *Honour* that is built upon it, consist in *Comparison* and *Preference*; and so what belongs to every body, belongs to no body; and because the *Rate* that is commonly set upon a Man, is taken from what he is able to do without the Assistance of others. (Hobbes, 1983: 1.2)

This is the form of esteem that some of Hobbes’s interpreters have taken to lead to competitiveness and status insecurity, both on the level of individuals (see Slomp, 2007) and on the level of international relations (see Piirimäe, 2006). On first sight, placing the discussion of esteem between peoples into this context, as Pufendorf does, suggests that something analogous could hold for Pufendorf’s views concerning the striving for esteem on the level of political communities. Things, however, are more complex.

In fact, Pufendorf is aware that matters of precedence are the object of intensive contention and that arguments from dependence play a crucial role in the practice of political argumentation:

[B]etween *Princes* and *Nations*, the Disputes about *Eminence* of *Worth* and *Dignity*, and the *Precendence* which depends upon it, have generally, and in all Ages, been managed with greater Show of Reason, and stronger Pretences . . . Thus much . . . is beyond Dispute, that where a *Prince* depends upon *Another* of *superior Power*, the greater is the most *honourable*, and has a *perfect Right* to *Precedence* . . . (1729: 810 [8.4.15])

As Pufendorf (1729) explains, relations of dependence can be feudal relations (perhaps the most pervasive type of dependence in early modern Europe), but he mentions other forms of dependence such as the strategy of ancient Rome to restrict local rulers to administrative tasks (810 [8.4.15]). A further type of dependence derives from unequal confederations: “[A]lso a *Prince* that has made an *unequal League* with another, doth by that very *Act* acknowledge a *Superiority* of *Worth* and *Dignity* or *Precedence*, either for his own *Person*, or as *Head* of such a *State* or *Commonwealth*, whether by *Compact* or *Custom* . . .” (1729: 810 [8.4.15]).

However, Pufendorf (1729) is highly critical of the idea of deriving honor from the position of one’s community in a web of dependence relations. As he notes, Hobbes “reduces all the *Foundations of Honour* or *intensive Esteem* to *Power*, which he calls a *Possession of present Means*, to *obtain some apparent future Good*” (p. 807 [8.4.13]; see Hobbes 2012: 10.16). Against Hobbes’s conception of worth or dignity of a person as “the value of price of a man, or as much as would be given for the use of his power” (2012: 10.16), Pufendorf (1729) objects that an opinion of power alone produces nothing but fear, and fear brings with it the wish of seeing the object of fear to be destroyed, which is the opposite of esteeming it (p. 808 [8.4.13]). In line with this objection, Pufendorf (1729) points out that in other passages Hobbes analyzes esteem as a combination of belief concerning power and belief concerning moral goodness (p. 808 [8.4.13]; see Hobbes, 1983: 15.9). Pufendorf (1729) suggests an analogous characterization of esteem between political communities: “The *Foundations* of *intensive Esteem* in general are all those Things which discover, or are supposed to imply, extraordinary Excellence or Perfection of any kind, the Effects of which are consonant to the *Laws of Nature*, and to the Ends of *Civil Government*” (p. 805 [8.4.12]).

According to this conception, not all of the things that “are apt to raise in other Men *Reverence* and *Respect*” are suitable of esteem between political communities. This does not imply that matters of power would become irrelevant for duties of esteem in international relations. One sense in which they remain relevant concerns question of political prudence: “As for *Power* indeed, that may often force the Weaker to pay the *Signs* of *Honour* to the Greater, because it is Madness not to yield to them that have Power to do us harm, when they please” (1729: 811 [8.4.18]). At the same time, Pufendorf rejects the idea that esteem between political communities should be proportional to means of power; rather, a degree of power sufficient for the fulfillment of the task of civil government is enough to reject claims to precedence:

And therefore, if one *Prince’s Territories* be six hundred Miles in Extent, and another’s but one hundred, yet the Difference in the Kingdoms makes none between the *Sovereigns*; for the Power is of the same Nature in the greater and in the less; and the *one* may answer the Ends of Government as well as the *other*. Not to say further, that *Power* alone, as it implies Ability to do Harm, doth not include any *Excellence* in it, which is naturally proper to command *sincere Respect*: For all *Respect* hath a Mixture of *Love* in it, but *Power* to do Harm can certainly produce nothing but *Hatred*. (1729: 812 [8.4.18])

Putting things in this way implies that better fulfillment of the ends of government can give precedence over more powerful political communities and their representatives. What grounds esteem for political communities and their representatives thus is not the use of power but rather their contribution to good governance. Fulfilling such duties of esteem is part of what natural law demands because it contributes to peaceful sociality.^7^

For this reason, Pufendorf believes that republics do not deserve less esteem in diplomatic relations than monarchies only for the reason that they may be less powerful:

[T]here is no *perfect Obligation*, which presupposes proper *Right* in another, by which a *Prince* really possessed of *sovereign Power* is bound to yield *Precedence* to any other *King* or *Prince* as more *honourable*, however *Superior* in the *Particulars* before mention’d; neither is one *free State* or *Commonwealth* obliged to yield more, though perhaps another is more antient, or more powerful. Nor indeed is a *State* govern’d by the *Populace*, inferior in Dignity to a *State* govern’d by a Prince, though in a *Democracy* there is no *particular* Person to be compar’d with a *King.* And hence an *Ambassador* from a *free Commonwealth* is not necessarily obliged to give *Precedence* to an *Ambassador* from a *crown’d Head*. (1729: 812 [8.4.20])

The only proviso that Pufendorf (1729) adds is that the envoys of republics have to yield precedence to princes because envoys have only “deriv’d” honor while the honor due to prince is “original” (p. 812 [8.4.20]). But this point seems to apply to all relations between individuals with representative character and bearers of sovereignty rights. This is why Pufendorf’s natural law-based account of duties of esteem in diplomatic relations could offer a solution to the problem of disesteem between political communities with different constitutional forms and different positions in the hierarchy of power.

## Esteem and the diplomacy of the Hanseatic cities

If esteem for political communities is mainly a function of their fulfillment of the goals of civil government, then why not extend esteem to *dependent* political communities? Independence is a matter of power, so restricting esteem to sovereigns that are defined by independence seems to concede too much to matters of power. Such a restriction of equal esteem would increase status uncertainty and the conflict potential that smaller powers pose for the established big powers. As far as I can see, these issues are never addressed in *De jure naturae et gentium*. However, some of the historical details given in Pufendorf’s history of the Swedish-German war suggest that his abstract considerations concerning duties of esteem in diplomatic relations could be applied to small communities such as the Hanseatic cities.

Pufendorf (1688) was clearly aware that the Hanseatic League fulfilled functions of the law of peoples through the role that they played in European diplomacy. For instance, he notes that, in 1627, Spain entered negotiations with the Hanseatic League over a trade alliance that would have given to Spanish merchants access to the Baltic sea and to Hanseatic merchants access to the Spanish market (1: 23); in 1628, the city of Stralsund sought the assistance of the Hanseatic cities to avert an attack of the imperial armies, and Hanseatic envoys tried (unsuccessfully) to negotiate a peaceful compromise (1688: 1: 36); in 1630, Hanseatic envoys tried (again unsuccessfully) to mediate between the city of Magdeburg and the imperial armies over the question of whether the city should provide winter quarters for the armies (1688: 1: 49); in 1635, the Hanseatic cities of Lübeck, Hamburg, Bremen and Braunschweig held a conference during which they accepted the Peace of Prague because their rights were protected by this agreement, and Pufendorf (1688) mentions an earlier separate alliance between Braunschweig and Sweden (1: 281); in 1641, envoys of Hanseatic cities (successfully) negotiated with high-ranking Swedish officers to defend their rights of continuing trade with goods that were not directly relevant for warfare—as they claimed, these rights are guaranteed by the law of peoples, and the Swedish officers complied because they had the realistic expectation that the Hanseatic League would be able to seek alliances with the king of Denmark or the Emperor should these demands be declined (1688: 1: 620). In 1645, the Hanseatic cities intervened (successfully) to spare Magdeburg from occupation either by troops of the king of Sweden or troops of the Emperor (1688: 2: 151); in 1646, France took the opposition of the Netherlands, the kings of Denmark and Poland, and the Hanseatic League against the impending transfer of Pomerania to Sweden as an argument in favor for speeding up the peace negotiations to prevent an alliances of the political entities opposing this increase of Swedish power (1688: 2: 238); and in 1648, the Hanseatic resident in the Netherlands played an active role in triggering opposition against including a provision concerning the right of the Counts of Oldenburg to raise tolls on traffic on the river Weser into a peace agreement (1688: 2: 484).

What is more, Pufendorf (1688) notes that claims to diplomatic representation on the ambassadorial level played an important, but contested role in the politics of the Hanseatic cities. Pufendorf reports that, in 1645, it was controversial whether the envoys of the Hanse should be admitted to the meetings of the ambassadors of the major powers (2: 189). The *Reichsstände* argued that, apart from the representation of those Hanseatic cities that were at the same time Imperial Cities, the Hanseatic League does not have a representation at the Imperial Diet and, hence, should be excluded from the peace negotiations. The Hanseatic cities rejoined that they need to be part of the peace negotiations because, like other parties in the war, they have restitution rights; and that these restitution rights are grounded in the violation of commercial rights that have been approved and confirmed both by the Empire and almost all European kings (1688: 2: 190). A year later, the German princes contested the claim to ambassadorial status made by the envoys of the Hanseatic League by arguing that some of the Hanseatic cities (those that are not Imperial Cities) are subjects of other powers (1688: 2: 259). This controversy over the status of the envoys of the Hanseatic cities is significant: Denying to the Hanseatic envoys expression of esteem that signalizes the recognition of the League’s ability to fulfill functions under the law of peoples created obstacles to the resolution of international conflicts because it would have been an obstacle to the full participation of the Hanseatic cities in peace negotiations. Even for dependent communities, Pufendorf’s historical observations indicate that recognizing the fulfillment of functions under the law of peoples would be highly desirable with a view to conflict resolution. And this is what, according to his natural law theory, grounds duties of esteem in diplomatic relations.

## Concluding remarks

Let me conclude with some brief remarks concerning why Leibniz’s and Pufendorf’s normative conceptions of duties of esteem could be thought-provoking even from a present-day perspective. Of course, relations of *feudal* dependence have disappeared from large parts of the planet; and certainly, we will be unmoved by the particular political agenda in which Leibniz was involved in the service of the house of the Guelfs. However, as we have seen, Pufendorf regards feudal law as a largely overcome relic from medieval times and analyses the relations within the German Empire mainly as relations of unequal confederation. And we have also seen that Leibniz takes the particular political controversy as an occasion to offer theoretical considerations concerning the nature of sovereignty and the ensuing duties of esteem that could have pushed him toward conclusions incompatible with his propagandistic goal. The present-day interest of Pufendorf’s and Leibniz’s considerations derives from their attention to two problems that have not disappeared from the planet: The general problem that a power-oriented conception of the self-worth of political communities leads to conflicts; and the special problem that this holds even for dependent communities and their representatives.

It is well documented how much status anxiety influences decisions in power politics (Paul et al., 2014), especially decisions concerning warfare (Lebow, 2010; Renshon, 2016; Wohlforth, 2009). What is more, it has been noted that, if status is understood to be a function of the position in a power hierarchy, it is almost impossible for small countries to engage in the activities that lead to status (Lebow, 2010: 200). In contemporary political theory, there are at least two approaches to how these problems could be solved. One approach derives from neo-Hegelian theories of unconditional recognition (as articulated, e.g. in Honneth, 1995, 2012; Wendt, 2003). Another approach derives from intercultural theories of international relations, according to which the desire for self-worth takes different forms in cultures focused on dignity, cultures focused on honor and cultures focused on saving face (Friedrichs, 2016; Lebow, 2008). Evidently, even giving an overview of these approaches would go beyond the limits of the present article. Still, it may be useful to indicate a few aspects that are distinctive about the approaches found in Leibniz and Pufendorf.

What sets Leibniz’s and Pufendorf’s approaches apart from theories of unconditional recognition is that assessing the degree to which communities fulfill functions of civil government is a comparative matter. Some communities will fare better and therefore deserve more esteem than others. But this is what is to be expected from the perspective of natural law theory: Communities that violate the demands of natural law should be disesteemed, not recognized, for this reason. At the same time, the duties of esteem in diplomatic relations that Leibniz and Pufendorf have in mind do not depend on success in the competition for power. Seeking esteem for the fulfillment of the goals of civil government, especially of functions in peace keeping and conflict resolution, may be a competitive affair—but competition in this sense can lead only to beneficial results and does not exclude smaller and dependent communities.

What sets Leibniz’s and Pufendorf’s approaches apart from intercultural theories is that they identify duties of esteem for political communities that do not derive from changes in cultural attitudes. To be sure, changes in the view of the nature of wealth and the acceptance of collective security architectures lead to status seeking on the basis of economic cooperation and participation in international institutions (Lebow, 2015). But access to equitable economic cooperation and full diplomatic representation in international institutions itself is a function of power—as witnessed by the many (often unsuccessful) initiatives of governments of developing countries or the Palestinian Authority. Cultural changes may in fact render competition for esteem between powerful nations less conflict-laden. But at the same time these changes may fail to provide a source of self-worth for smaller, and especially for dependent communities. The applicability of Leibniz’s and Pufendorf’s approaches to small and dependent communities may offer a response to these concerns. Leibniz and Pufendorf provide reasons for why small and dependent communities could make legitimate claims to full representation in international negotiations. This reverts the direction of argument: Status should not be understood to be a function of representation in international institutions; rather, representation on the ambassadorial level should be understood to be a function of the ability to fulfill tasks of civil government.

## Notes

1. For detailed analysis, see Randelzhofer, 1967; Von Aretin, 1986.

2. On Pufendorf’s analysis of the constitution of the German Empire, see Döring, 1994; Schröder, 1999.

3. On the genesis of this text, see Döring, 1994.

4. On Pufendorf on federalism, see Defour, 1996.

5. See Leibniz’s references to Pufendorf’s *De statu imperii Germanici* in Leibniz, 1923: IV, 2: 65; 67; 114; 275, note 3; 291, note 1; 488, note 53; on Leibniz’s analysis of the constitution of the German Empire, see Hammerstein, 1974; Piro, 2011.

6. On this provision of the peace of Westphalia, see Böckenförde, 1969.

7. On Pufendorf on sociality, see Saastamoinen, 2019.

## References

[bibr1-17550882211002225] Böckenförde E-W (1969) Der Westfälische Frieden und das Bündnisrecht der Reichsstände. Der Staat 8: 449–478.

[bibr2-17550882211002225] Bodin J (1576) Six livres de la république. Paris: Du Puys.

[bibr3-17550882211002225] Conring H Bode G (1652) Exercitatio de urbibus Germanicis. Helmstedt: Müller.

[bibr4-17550882211002225] de Monzambano S [Pufendorf S] (1667) De statu imperii Germanici. Geneva: Colomesius.

[bibr5-17550882211002225] Defour A (1996) Pufendorfs föderalistisches Denken und Staatsräsonlehre. In: Palladini F Hartung G (eds) Samuel Pufendorf und die europäische Frühaufklärung. Berlin: Akademie Verlag, pp.105–122.

[bibr6-17550882211002225] Döring D (1994) Untersuchungen zur Entstehungsgeschichte der Reichsverfassungsschrift Samuel Pufendorfs. Der Staat 33(1): 185–206.

[bibr7-17550882211002225] Friedrichs J (2016) An intercultural theory of international relations. International Theory 8(1): 63–96.

[bibr8-17550882211002225] Haara H (2018) Pufendorf’s Theory of Sociability: Passions, Habits and Social Order. Cham: Springer.

[bibr9-17550882211002225] Haara H Lahdenranta A (2018) Smithian sentimentalism anticipated: Pufendorf on the desire for esteem and moral conduct. Journal of Scottish Philosophy 16(1): 19–37.

[bibr10-17550882211002225] Haara H Stuart-Buttle T (2019) Beyond justice: Pufendorf and Locke on the desire for esteem. Political Theory 47(5): 699–723.

[bibr11-17550882211002225] Hammerstein N (1974) Leibniz und das Heilige Römische Reich deutscher Nation. Nassauische Annalen 85(1): 87–102.

[bibr12-17550882211002225] Henniges H (1687) Discursus de suprematu adversus Caesarinum Furstenerium. Hyetopoli ad Istrum [no publisher].

[bibr13-17550882211002225] Hobbes T (1983) De cive. Oxford: Clarendon Press.

[bibr14-17550882211002225] Hobbes T (2012) Leviathan. Oxford: Clarendon Press.

[bibr15-17550882211002225] Honneth A (1995) The Struggle for Recognition: The Moral Grammar of Social Conflicts. Cambridge: Polity.

[bibr16-17550882211002225] Honneth A (2012) The I in We: Studies in the Theory of Recognition. Cambridge: Polity.

[bibr17-17550882211002225] Hruschka J (2000) Existimatio: Unbescholtenheit und Achtung vor dem Nebenmenschen bei Kant und in der Kant vorangehenden Naturrechtslehre. Jahrbuch für Recht und Ethik 8(1): 181–195.

[bibr18-17550882211002225] Lebow RN (2008) An Intercultural Theory of International Relations. Cambridge: Cambridge University Press.

[bibr19-17550882211002225] Lebow RN (2010). Why Nations Fight: Past and Future Motives for War. Cambridge: Cambridge University Press.

[bibr20-17550882211002225] Lebow RN (2015). *Thumos*, War, and Peace. Common Knowledge 21: 50–82.

[bibr21-17550882211002225] Leibniz GW (1923—) Sämtliche Schriften und Briefe. Darmstadt, Leipzig and Berlin: Akademie-Verlag.

[bibr22-17550882211002225] Paul TV Larson DW Wohlforth WC (eds) (2014). Status in World Politics. New York: Cambridge University Press.

[bibr23-17550882211002225] Piirimäe P (2006) The explanation of conflict in Hobbes’s Leviathan. Trames 10(1): 3–21.

[bibr24-17550882211002225] Piro F (2011) Rationality of the irregular. Political communities and constitutional devices in Leibniz. Studia Leibnitiana 43(1): 36–53.

[bibr25-17550882211002225] Pufendorf S (1684) De jure naturae et gentium, 2nd edn. Frankfurt: Knochius.

[bibr26-17550882211002225] Pufendorf S (1688) Sechs und Zwantzig Bücher der Schwedisch- und Deutschen Kriegsgeschichte. Frankfurt and Leipzig: Gleditsch.

[bibr27-17550882211002225] Pufendorf S (1729) Of the Law of Nature and Nations (trans. Kennett B ), 4th edn. London: Walthoe, Wilkin, Bonwicke, Birt, Ward & Osborne.

[bibr28-17550882211002225] Randelzhofer A (1967) Völkerrechtliche Aspekte des Heiligen Römischen Reiches nach 1648. Berlin: Duncker & Humblot.

[bibr29-17550882211002225] Renshon J (2016) Status deficits and war. International Organization 70(3): 513–550.

[bibr30-17550882211002225] Saastamoinen K (2010) Pufendorf on Natural equality, human dignity, and self-esteem. Journal of the History of Ideas 71(1): 39–62. 20503631 10.1353/jhi.0.0065

[bibr31-17550882211002225] Saastamoinen K (2019) Pufendorf on the law of sociality and the law of nations. In: Zurbuchen S (ed.) The Law of Nations and Natural Law, 1625-1850. Leiden: Brill, pp.107–131.

[bibr32-17550882211002225] Schröder P (1999) The constitution of the holy roman empire after 1648. Samuel Pufendorf’s assessment in his *Monzambano*. Historical Journal 42(4): 961–983.

[bibr33-17550882211002225] Slomp G (2007) Hobbes on glory and civil strife. In: Springborg P (ed.) The Cambridge Companion to Thomas Hobbes. Cambridge: Cambridge University Press, pp.181–198.

[bibr34-17550882211002225] von Aretin KO (1986) Das alte Reich 1648-1806. Band 1: Föderalistische oder hierarchische Ordnung (1648-1684). Stuttgart: Klett-Cotta.

[bibr35-17550882211002225] Wendt A (2003) Why a world state is inevitable. European Journal of International Relations 9(4): 491–542.

[bibr36-17550882211002225] Wohlforth WC (2009). Unipolarity, status competition, and great power war. World Politics 61(1): 28–57.

